# Supplementary material for: DNA barcoding indicates multiple invasions of the freshwater snail Melanoides tuberculata sensu lato in Florida
Source: PLoS One. 2023 Dec 22;18(12):e0292164. doi: 10.1371/journal.pone.0292164 (PMC10745163; doi:10.1371/journal.pone.0292164)
Supplement: Attachment — Submitted filename: PONE-D-22-27150 rev.pdf [file pone.0292164.s002.pdf]

PLOS ONE

DNA barcoding indicates multiple invasions of the freshwater snail Melanoides tuberculata in Florida.

--Manuscript Draft--

Manuscript Number:	PONE-D-22-27150
Article Type:	Research Article
Full Title:	DNA barcoding indicates multiple invasions of the freshwater snail Melanoides tuberculata in Florida.
Short Title:	Melanoides tuberculata in Florida
Corresponding Author:	Michael A Chadwick, PhD<br>King's College London<br>London, UNITED KINGDOM
Keywords:	Thiaridae; shell form; haplotype; invasive species; phylogenetic
Abstract:	Melanoides tuberculata is a tropical, aquatic snail of Asian and African origin that has successfully invaded freshwaters worldwide, including Florida, USA. In general, these snails are female clonal, resulting in populations with unique haplotypes and shell forms. To understand patterns of dispersal of snails in Florida, mitochondrial DNA (cytochrome oxidase I) was sequenced to determine the number of haplotypes and the region of origin (Asia or Africa) through phylogenetic reconstruction including sequences from globally distributed snails. In total, 9 haplotypes were recovered, and phylogenetic reconstruction yielded a topology with two distinct clades. One clade was most closely related to individuals from west Asia (Iran) including one Florida-specific population and the other was closely related to individuals from southeast Asia (Malaysia and Thailand). Additionally, we looked at shell morphology in relation to haplotypes and found that multiple haplotypes were represented by similar shell forms with only two morphs being represented by unique haplotypes. Overall, no spatial correlations of haplotypes, region of origin, or shell morphs were recovered. These results suggest that independent introductions are likely the primary mechanism of dispersal of M. tuberculata in Florida.
Order of Authors:	Lori R. Tolley-Jordan
	Michael A Chadwick, PhD
	Jimmy Triplett
Additional Information:	
**Question**	**Response**
**Financial Disclosure**<br><br>Enter a financial disclosure statement that describes the sources of funding for the work included in this submission. Review the submission guidelines for detailed requirements. View published research articles from *PLOS ONE* for specific examples.<br><br>This statement is required for submission and **will appear in the published article** if the submission is accepted. Please make sure it is accurate.	Funding was provided by United States Fish and Wildlife Service, Gulf States Marine Fisheries<br>Division (project ID: FWS-800-037-2016-JSU) and the McCluer Scholar Award given to L.<br>Tolley-Jordan by the School of Science, Jacksonville State University

Powered by Editorial Manager® and ProduXion Manager® from Aries Systems Corporation

**Unfunded studies**<br>Enter: *The author(s) received no specific funding for this work.*<br><br>**Funded studies**<br>Enter a statement with the following details:<br>• Initials of the authors who received each award<br>• Grant numbers awarded to each author<br>• The full name of each funder<br>• URL of each funder website<br>• Did the sponsors or funders play any role in the study design, data collection and analysis, decision to publish, or preparation of the manuscript?<br>• **NO** - Include this sentence at the end of your statement: *The funders had no role in study design, data collection and analysis, decision to publish, or preparation of the manuscript.*<br>• **YES** - Specify the role(s) played.<br><br>* typeset	
**Competing Interests**<br><br>Use the instructions below to enter a competing interest statement for this submission. On behalf of all authors, disclose any competing interests that could be perceived to bias this work—acknowledging all financial support and any other relevant financial or non-financial competing interests.<br><br>This statement is required for submission and **will appear in the published article** if the submission is accepted. Please make sure it is accurate and that any funding sources listed in your Funding Information later in the submission form are also declared in your Financial Disclosure statement.<br><br>View published research articles from *PLOS ONE* for specific examples.	The authors have declared that no competing interests exist.

**NO authors have competing interests**<br><br>Enter: *The authors have declared that no competing interests exist.*<br><br>**Authors with competing interests**<br><br>Enter competing interest details beginning with this statement:<br><br>*I have read the journal's policy and the authors of this manuscript have the following competing interests: [insert competing interests here]*<br><br>* typeset	
**Ethics Statement**<br><br>Enter an ethics statement for this submission. This statement is required if the study involved:<br><br>• Human participants<br>• Human specimens or tissue<br>• Vertebrate animals or cephalopods<br>• Vertebrate embryos or tissues<br>• Field research<br><br>Write "N/A" if the submission does not require an ethics statement.<br><br>General guidance is provided below. Consult the submission guidelines for detailed instructions. **Make sure that all information entered here is included in the Methods section of the manuscript.**	N/A

Powered by Editorial Manager® and ProduXion Manager® from Aries Systems Corporation

**Format for specific study types**<br><br>**Human Subject Research (involving human participants and/or tissue)**<br>• Give the name of the institutional review board or ethics committee that approved the study<br>• Include the approval number and/or a statement indicating approval of this research<br>• Indicate the form of consent obtained (written/oral) or the reason that consent was not obtained (e.g. the data were analyzed anonymously)<br><br>**Animal Research (involving vertebrate animals, embryos or tissues)**<br>• Provide the name of the Institutional Animal Care and Use Committee (IACUC) or other relevant ethics board that reviewed the study protocol, and indicate whether they approved this research or granted a formal waiver of ethical approval<br>• Include an approval number if one was obtained<br>• If the study involved *non-human primates*, add *additional details* about animal welfare and steps taken to ameliorate suffering<br>• If anesthesia, euthanasia, or any kind of animal sacrifice is part of the study, include briefly which substances and/or methods were applied<br><br>**Field Research**<br><br>Include the following details if this study involves the collection of plant, animal, or other materials from a natural setting:<br>• Field permit number<br>• Name of the institution or relevant body that granted permission	
**Data Availability**<br><br>Authors are required to make all data underlying the findings described fully available, without restriction, and from the time of publication. PLOS allows rare exceptions to address legal and ethical concerns. See the PLOS Data Policy and FAQ for detailed information.	Yes - all data are fully available without restriction

Powered by Editorial Manager® and ProduXion Manager® from Aries Systems Corporation

A Data Availability Statement describing where the data can be found is required at submission. Your answers to this question constitute the Data Availability Statement and **will be published in the article**, if accepted.<br><br>**Important:** Stating 'data available on request from the author' is not sufficient. If your data are only available upon request, select 'No' for the first question and explain your exceptional situation in the text box.<br><br>Do the authors confirm that all data underlying the findings described in their manuscript are fully available without restriction?	
**Describe where the data may be found in full sentences. If you are copying our sample text, replace any instances of XXX with the appropriate details.**<br><br>• If the data are **held or will be held in a public repository**, include URLs, accession numbers or DOIs. If this information will only be available after acceptance, indicate this by ticking the box below. For example: *All XXX files are available from the XXX database (accession number(s) XXX, XXX.*).<br>• If the data are all contained **within the manuscript and/or Supporting Information files**, enter the following: *All relevant data are within the manuscript and its Supporting Information files.*<br>• If neither of these applies but you are able to provide **details of access elsewhere**, with or without limitations, please do so. For example:<br><br>*Data cannot be shared publicly because of [XXX]. Data are available from the XXX Institutional Data Access / Ethics Committee (contact via XXX) for researchers who meet the criteria for access to confidential data.*<br><br>*The data underlying the results presented in the study are available from (include the name of the third party*	Relevant data are within the manuscript and its Supporting Information files. Additionally, all sequence data have been uploaded to GenBank.

Powered by Editorial Manager® and ProduXion Manager® from Aries Systems Corporation

and contact information or URL).<br>• This text is appropriate if the data are owned by a third party and authors do not have permission to share the data.<br><br>* typeset	
Additional data availability information:	Tick here if the URLs/accession numbers/DOIs will be available only after acceptance of the manuscript for publication so that we can ensure their inclusion before publication.

Powered by Editorial Manager® and ProduXion Manager® from Aries Systems Corporation

Manuscript

Click here to access/download;Manuscript;PLOS_FLASNAIL_30SEPT_FINAL

DNA barcoding indicates multiple invasions of the freshwater snail *Melanoides tuberculata* in Florida.

Lori R. Tolley-Jordan[1] Michael A. Chadwick[2*] and Jimmy Triplett[1]

[1]Dept. of Biology, Jacksonville State University, Jacksonville, AL, USA

[2]Dept. of Geography, King's College London, London, UK

* Corresponding author e-mail: michael.chadwick@kcl.ac.uk

ORCID: LRTJ: 0000-0002-9195-1252; MAC: 0000-0003-4891-4357; JT: 0000-0002-2381-4168

Abstract

Melanoides tuberculata is a tropical, aquatic snail of Asian and African origin that has successfully invaded freshwaters worldwide, including Florida, USA. In general, these snails are female clonal, resulting in populations with unique haplotypes and shell forms. To understand patterns of dispersal of snails in Florida, mitochondrial DNA (cytochrome oxidase I) was sequenced to determine the number of haplotypes and the region of origin (Asia or Africa) through phylogenetic reconstruction including sequences from globally distributed snails. In total, 9 haplotypes were recovered, and phylogenetic reconstruction yielded a topology with two distinct clades. One clade was most closely related to individuals from west Asia (Iran) including one Florida-specific population and the other was closely related to individuals from southeast Asia (Malaysia and Thailand). Additionally, we looked at shell morphology in relation to haplotypes and found that multiple haplotypes were represented by similar shell forms with only two morphs being represented by unique haplotypes. Overall, no spatial correlations of haplotypes, region of origin, or shell morphs were recovered. These results suggest that independent introductions are likely the primary mechanism of dispersal of *M. tuberculata* in Florida.

Keywords: Thiaridae, shell form, haplotype, invasive species, phylogenetic

Introduction

Melanoides tuberculata (Müller 1774) is a freshwater snail endemic to Africa and Asia but in the past century it has successfully invaded warm-water systems (see review in [1]). The ability of individuals of *M. tuberculata* to survive a wide range of stressors [2] including ones associated with dispersal such as desiccation [3], salinity [4], and high temperatures [5] are central factors for its success in colonizing novel habitats. Unfortunately, due to the ubiquitous presence of these snails in the aquarium trade, human-mediated spread is likely the main invasion pathway [6–9]. Moreover, these snails have parthenogenetic, iteroparous reproduction and generalist feeding strategies [2,10] allowing invasive populations to reach densities of >100 individuals per m^2 [11–13]. These traits likely facilitated secondary spread of snails in invaded regions. However, independent invasion event associated with aquarium dumping has been suggested to be the primary cause of range expansions [8,14].

Although the native range of *M. tuberculata* is expansive (e.g., East Africa to Southeast Asia), populations in these regions are not homogenous [10,15,16] as each region has distinct clades with unique haplotypes [17,18]. However, invasive populations are thought to be primarily female with limited sexual reproduction [19], suggesting that population genetics are conserved and provide useful evidence to track both primary invasion and secondary spread. Indeed, the use of mitochondrial (cytochrome c oxidase I) and ribosomal markers (16s rDNA) have been highly successful in understanding patterns of origin associated with *M. tuberculata* [8–10,16–18]

Along with molecular phylogenetics, haplotype analysis for *M. tuberculata* has been useful for evaluating invasion patterns. Haplotypes from close proximities are more similar than from distant regions (e.g., African lineages are more similar to each other than Southeast Asia)

and this has allowed studies to verify the geographic origins of invasive lineages [10,17,18]. Additionally, some studies have shown that unique haplotypes are typically represented by a unique shell morph [10,17,18,20] which suggests that shell morphometric analyses could be used to evaluate invasion dynamics. To date, this information has been used to verify that invasive lineages in the New World are from Southeast Asia and that African lakes were invaded by individuals from Southeast Asia [10,17], as a well-supported clade including individuals from invaded and native areas is indicative of a single invasive origin [10,17]. However, shell form analyses used to determine distinct morphs can be more difficult to interpret as some lineages from Southeast Asia have phenotypically plastic shell forms [10,15]. Further, morphs unique to invaded areas can occur, although these may be a result of hybridization as shown in the Caribbean Island of French Martinique [20]. Regardless, the combination of genetics and morphological evaluations of *M. tuberculata* populations provide evidence regarding both geographic regions of origin and putative invasion pathways.

The focus of this study was to evaluate if invasive *M. tuberculata* in Florida, USA are dispersed primarily through independent invasions, range expansion of populations through connected waterways, or a combination of both. Populations of *M. tuberculata* were first documented in the 1960s and subsequently, over 45 locations have been identified where *M. tuberculata* was present [21,22]. The distribution is suggested to be state-wide with snails living in a variety of water bodies such as ponds, springs, and canals. Although aquarium release is the suspected mechanism for the founding population in the Middle River and Florida Canals near Ft. Lauderdale, Florida [23], it is unknown if other established populations in Florida arose from aquarium releases or via natural range expansion across the many inter-connected waterways in the state. In the literature, only one population with a singular morph from Florida has been

included in phylogenetic and morphological analyses [10] and invasion pathways are not well understood. As such, to address the goals of the study we use molecular and morphological data to explore putative invasion pathways of *M. tuberculata* in Florida waterways. Specifically, we evaluated geographic regions of origin through phylogenetic reconstruction and determined the number and distribution of haplotypes across the state using a barcoding gene marker, cytochrome c oxidase I (CO1). Additionally, we used established morphometric indices to determine the numbers of shell morphs, their dispersion across the state, and their relationship with observed haplotypes.

Methods:

Snail Collections

We surveyed 25 waterbodies throughout Florida [24]. From each site where we live snails were found, we aimed to collect 10 individuals for molecular analyses. From all individuals, head tissues were removed and preserved in 95% molecular grade ethanol. In addition, tissues from 4 individuals of *Tarebia granifera* collected from Florida were used as an outgroup in our phylogenetic analyses. All preserved snails were vouchered at the Jacksonville State University invertebrate collection.

DNA Sequencing

DNA was extracted and isolated from head tissue using 20uL proteinase K with 180µL ATL buffer from a Qiagen DNeasy Blood and Tissue Kit and the process was completed using a Qiagen Plant Extraction Kit. We used a combined method for DNA extraction because polysaccharides found in the snail tissue act as a polymerase chain reaction (PCR) inhibitor [25].

A fragment of the mitochondrial cytochrome c oxidase I (COI) gene was amplified using the forward primer LCO1490 (5’-GGTCAACAAATCATAAAGATATTGG-3’) and the reverse primer HCO2198 (5’-TAAACTTCAGGGTGACCAAAAAATCA-3’; [26]) in polymerase chain reactions (PCR) carried out in 25 µL volumes using 12.5 µL of BioMix Red (Bioline, Taunton, MA), 2.5 µL (10 mM) of the forward and reverse primers described above, 5 µL of sterile deionized water, and 5µL of DNA. PCR was performed in a thermocycler using a cycling profile of initial denaturation at 95 ˚C for one minute and 35 cycles of denaturation at 95 ˚C for one minute, annealing at 56 ˚C for one minute, and elongation at 72 ˚C for 90 seconds followed by a final elongation step at 72 ˚C for seven minutes. Amplicons were confirmed on a 1% agarose gel stained with SYBR Green (ThermoFisher Scientific). Amplicons were cleaned using ExoSAPIT (Affymetrix) following the manufacturer’s protocol. DNA sequencing was completed at Molecular Cloning Laboratories (MCLAB; South San Francisco, CA). All individual sequences from this study are available in NCBI: GenBank (*Tarebia granifera*: MT671805-MT671808 and *M. tuberculata*: OP114776 - OP11824).

Genetic Diversity of Florida Snails

CO1 sequences ranging from 570 to 641 bp of 49 individuals from 11 sampled locations were assembled, verified, and aligned using the MUSCLE algorithm in MEGA X [27]. This subset of data was subsequently referred to as ‘Florida’ (see supplementary materials for GenBank accession numbers). Haplotype diversity (*h*) and nucleotide diversities (π) were produced in DnaSP 6.12.03 [28]. A Mantel test was conducted on a Tamura- Nei distance matrix of derived haplotypes for each sampling location (generated in MEGA X) to determine the correlation between geographic distance and haplotype.

Lineage Origin of Florida Snails

To evaluate the lineage origin of Florida snails we assembled COI snail sequences from Africa, the Indian Ocean, the Middle East, and Southeast Asia available on GenBank. These sequences were aligned using the MUSCLE algorithm in MEGA X [27]. This subset of data was subsequently referred to as 'World'. To verify the potential geographic origins of the Florida snails, the 'Florida' and 'World' subsets were combined and the number of polymorphic sites, number of haplotypes, haplotype diversity (hd), and nucleotide diversities (π) of the 'World' (n =36), 'Florida' (n = 49), and total sequences (n =85) were calculated. Genetic differentiation between the 'Florida' and 'World' sequences was determined using a Chi-square analysis. All analyses were conducted in DnaSP 6.12.03 [28].

The final data set included 46 sequences with 586 characters which included the 36 world haplotypes, nine Florida haplotypes, and one haplotype of *T. granifera* collected from site 15 that served as the outgroup. The 46 aligned sequences were analysed with maximum parsimony (MP) that resulted in 116 parsimony informative characters. All analyses were conducted in PAUP* [33]. Node stability was determined by bootstrap resampling of 1000 replicates of heuristic searches. Parsimony analyses resulted in 28 retained topologies with a consistency index of 0.7701 and a retention index of 0.9615. The strict consensus tree generated from this analysis was used as the final topology. The data set was also analysed using Bayesian inference (BI) with MrBayes 3.2 [29]. BI analyses were conducted using a partitioned GTR + I + G model for reasons outlined by Huelsenbeck and Rannala [30], with all parameter values estimated during analysis. A Dirichlet prior was used for base frequencies and the rate matrix. A uniform prior was used for the shape parameter (α), proportion of invariable sites (I), and topology. Branch

lengths were unconstrained. Partitions were designated for each data set and for the microstructural characters and all parameters were unlinked across partitions. Four separate MCMC runs were initiated, each with 10,000,000 generations. Runs were started from a random tree; the topology was sampled every 1,000 generations of the MCMC chain. Majority rule (50%) consensus trees were constructed after removing the first 10% of sampled trees ("burn-in"). Branch support was assessed according to a 0.95 posterior probability measure for BI [31,32]. The lineage origin from the 36 Genbank sequences were listed by the author, except for sequences from Iran.

Shell Morphology

Before evaluating morphological features on shells, specimens were cleaned with soap and water and a soft, bristle brush to remove deposits. Cleaning was done delicately to not damage shell features. We used the scoring system developed by Facon et al. [17] modified by Genner et al. [34] and Van Bocxlaer et al. [10] to determine shell morphs. All scores listed as not applicable were assigned a score of zero in the analyses. All sequenced shells were evaluated. Shells with the same scores were then grouped resulting in discrete shell morphs. To evaluate overall differences in Florida shell morphs, we used the character matrix of each discrete morph (Table 4) and non-dimensional scaling analyses (NDS) conducted in PAST v.4.03 [35].

Results

From the 25 locations sampled 11 were found to have live snails (Table 1, [24]). Approximately 350 individuals were collected across the 11 sampling locations. At four sites fewer than the target of 10 snails for genetic analyses were collected (sites 1, 6, and 24 = 6 individuals; site 3 =

2 individuals). At these sites, only individuals less than 8 mm were collected, and these individuals were not included in morphological analyses

Table 1. Sampling locations where live snails were collected. # Sequenced are the total number of individuals successfully sequenced at each site. Nine discrete haplotypes were identified using DnaSP 6.12.03 (Rozas et al. 2017). These data represent the ‘Florida’ dataset.

Site	Latitude	Longitude	Water Body	City	# sequenced	Haplotypes
Site 1	28.0744	-82.3664	Retention Pond	Tampa	4	*e*
Site 3	28.0487	-82.2685	Baker Creek	Tampa	7	*h*
Site 4	27.5273	-82.5132	Trib. to Manatee River	Ellinton	4	*g, h*
Site 6	25.4705	-80.3716	Trib. to Florida City Canal	Homestead	1	*b*
Site 8	25.4485	-80.4199	Florida City Canal	Homestead	4	*d*
Site 13	26.2309	-80.1522	Pompano Canal	Pompano Beach	6	*g, i*
Site 15	26.1645	-80.2450	Middle River Canal	Sunrise	3	*f, g*
Site 19	28.1346	-80.6714	Eau Galle Creek	Melbourne	4	*a, c, g*
Site 21	29.1680	-81.3004	Dias Lake	DeLeon Springs	7	*a*
Site 24	28.3967	-80.6114	Retention pond	Cocoa Beach	1	*e*
Site 25	30.2784	-81.6437	Trib. to New Rose Creek	Jacksonville	8	*e*

Genetic Diversity of Florida Snails

We sequenced 1-8 snails per sample location (Table 1). In total, nine haplotypes from ‘Florida’ were recovered. Three haplotypes (*a*, *e*, and *g*) were the most common and found in 39 individuals in 8 out of 11 sampling locations (Table 1, Fig. 1). More specifically, haplotype *g* was found at 4 sites, haplotype *e* was found at 3 sites and haplotype *a* at 2 sites. At four sites more than one haplotype was found (Table 1). The site with the greatest haplotype diversity was 19 with three haplotypes, while sites 4 and 15 each had two haplotypes. Only sites 6 and 8 had private haplotypes (Table 2). The Mantel test failed to show a correlation between site location and haplotype occurrence ($r = -0.0092$, $p = 0.417$).

Table 2. Accessioned sequences from NCBI: GenBank included in analyses. *Origin* indicates the country where the snail was collected (Africa- AF, Indian Ocean- IO, Middle East-ME, Southeast Asia- SEA). *Status* indicates if snails were considered Native or Invasive (based on GenBank metadata); ***** indicated origins that were available via GenBank metadata but assumed based on our phylogenetic tree. These data represent the 'World' dataset. N/A = data not available. DRC = Democratic Republic of the Congo.

Genbank	NA	Waterbody	Origin	Status
AY456563	N/A	Lake Malawi	N/A	Invasive*
AY456564	N/A	Lake Malawi	N/A	Invasive*
AY575971	Thailand	N/A	SEA	Native
AY575974	Singapore	Pandan Reservoir	SEA	Native
AY575976	Singapore	Upper Selatar	SEA	Native
AY575978	Singapore	Selatar River	SEA	Native
AY575979	Singapore	Lower Selatar	SEA	Native
AY575982	Mayotte	N/A	IO	Native
AY575985	Malawi	Lake Malawi	SEA	Invasive
AY575986	Malawi	Lake Malawi	SEA	Invasive
AY575990	Malawi	Lake Malombe	SEA	Invasive
AY575991	Malawi	Lake Malombe	SEA	Invasive
AY575992	Malawi	Lake Malawi	SEA	Invasive
AY575993	Malawi	Lake Malawi	SEA	Invasive
AY575994	Israel	N/A	ME	Native
AY575995	Tanzania	Lake Victoria	AF	Native
KP284130	Malaysia	N/A	SEA	Native
KP284131	Malaysia	N/A	SEA	Native
KP284132	Malaysia	N/A	SEA	Native
KP284133	Malaysia	N/A	SEA	Native
KP284134	Malaysia	Endau River	SEA	Native
KP284135	Malaysia	Sarawak River	SEA	Native
KP284136	Malaysia	N/A	SEA	Native
KP284137	Malaysia	Lenek River	SEA	Native
KP284138	Malaysia	Besuit River	SEA	Native
KP284139	Malaysia	Pahang River	SEA	Native
KP774674	Burundi	Lake Tanganyika	SEA	Invasive
KP774687	DRC	Makiso Stream	SEA	Invasive
KP774701	DRC	Lake Edward	AF	native
KP774703	DRC	Semliki River	AF	native
KP774705	DRC	Semliki River	SEA	Invasive
KP774710	Malawi	Lake Malawi	SEA	Invasive
KP774711	Malawi	Lake Malawi	SEA	Invasive
KT280408	Iran	N/A	ME	Native*
KT280409	Iran	N/A	ME	Native*
KT280412	Iran	N/A	ME	Native*

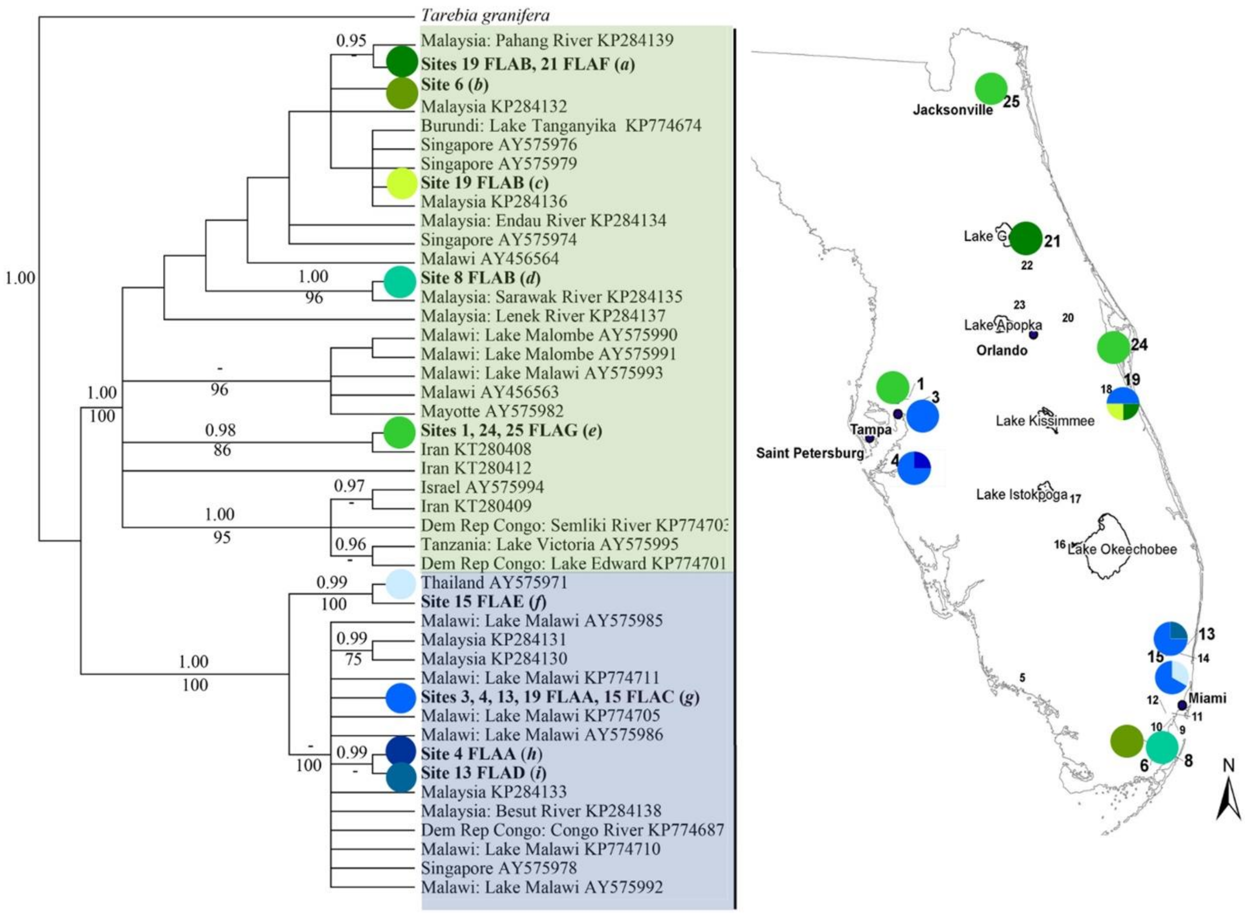

Figure 1. (Left) Maximum parsimony strict consensus tree of CO1 sequences from 'World' (Genbank, n = 35) and 'Florida' (n = 49). Bayesian scores greater equal to 0.95 above branches and parsimony informative scores >75 below. 'World' sequences are indicated by locality and Genbank accession number. 'Florida' sequences are identified by sampling location and shell morph. Haplotypes are identified by letter and corresponding color code. (Right) Locations of sampling sites in Florida, USA. Pie diagrams indicate the frequency of haplotypes at each sampling site. Haplotype colors correspond to the left panel.

Geographic regions of origin

We identified 36 sequences from Genbank to evaluate the geographic region of origin for the 'Florida' snails (Table 2, Fig. 1); i.e. 'World' dataset). In total 28 unique haplotypes were identified (Table 3). Seven of the 'Florida' haplotypes were identical to haplotypes found in the 'World' subset. 'Florida' haplotypes *h* and *i* were unique relative to sequences found in the 'World' subset. This finding most likely accounts for the significant differentiation between

'Florida' and the 'World' ($\chi^2 = 50.899$, $p < 0.001$). Overall, sequences from Florida represented about 32% of the haplotype diversity found in 'World'. Nucleotide diversity was similar between 'Florida' and 'World' (Table 3).

Table 3. Genetic variation of CO1 data from GenBank sequences (World) sequences from this study (Florida), and the entire dataset based on numbers of polymorphic sites, haplotype number, haplotype diversity (H_d ± Standard Error) and nucleotide diversity (π ± Standard Error).

Location	n	polymorphic sites	number haplotypes	H_d ± SE	π ± SE
World	36	128	28	0.975 ± 0.002	0.080 ± 0.004
Florida	49	112	9	0.739 ± 0.001	0.080 ± 0.000
Total	85	134	30	0.881 ± 0.024	0.081 ± 0.002

Phylogenetic reconstruction of the combined dataset (i.e. 'Florida and 'World') resulted in two distinct clades statistically supported by both Parsimony and Bayesian analyses (Fig. 1). Clade 1 included five Florida haplotypes and sequences associated with Africa, the island of Mayotte in the Indian Ocean, the Middle East, and Southeast Asia, along with individuals native to Southeast Asia that invaded Lakes Malawi, Malombe, and Tanganyika. Clade 1 split into two distinct subclades: one subclade included 'Florida' haplotypes *a*, *b*, *c*, *d* as well as sequences from Southeast Asia; the other subclade include Florida haplotype *e* as well as sequences from Africa, the Indian Ocean, and the Middle East (Fig. 1). Clade 2 included taxa native to Southeast Asia, and ones that invaded Lake Malawi and the Congo River. This clade also included Florida haplotypes *f*, *g*, *h*, *i*. Two unique 'Florida' haplotypes were included in Clade 2. These haplotypes are assumed to be of Southeast Asian origin based on strong evidence from phylogenetic reconstruction (Fig. 1).

Shell Morphology

Shell character analyses resulted in seven distinct morphs (NDS stress = 0.06; Fig. 2, Table 4). Both clades had unique shell morphs (Fig. 1.; i.e., no shell morph occurred in both clades 1 and 2). Individual shell morphs were not always represented by a single haplotype. For example, morphs FLAA and FLAC were both represented as haplotype *g* (clade 2), while morphs FLAB and FLAF were associated with haplotype *a* (clade 1). Alternatively, a single morph could represent multiple haplotypes. This was seen for morph FLAB represented haplotype *a* at site 19 and haplotype *d* at site 8. Additionally, this was the case for FLAA found at sites 3, 4, 12, and 19 which were associated with haplotype *g* and site 4 associated with haplotype *h.* Further, FLAB was restricted to Clade 1 but represented three haplotypes at sites 8 (haplotype *d*) and 19 (haplotypes *a* and *c*). Only morphotypes FLAE and FLAD were restricted to a single haplotype (Fig. 1, Table 3). These results clearly show a disconnect between shell morphology, haplotype, and overall genetic lineage.

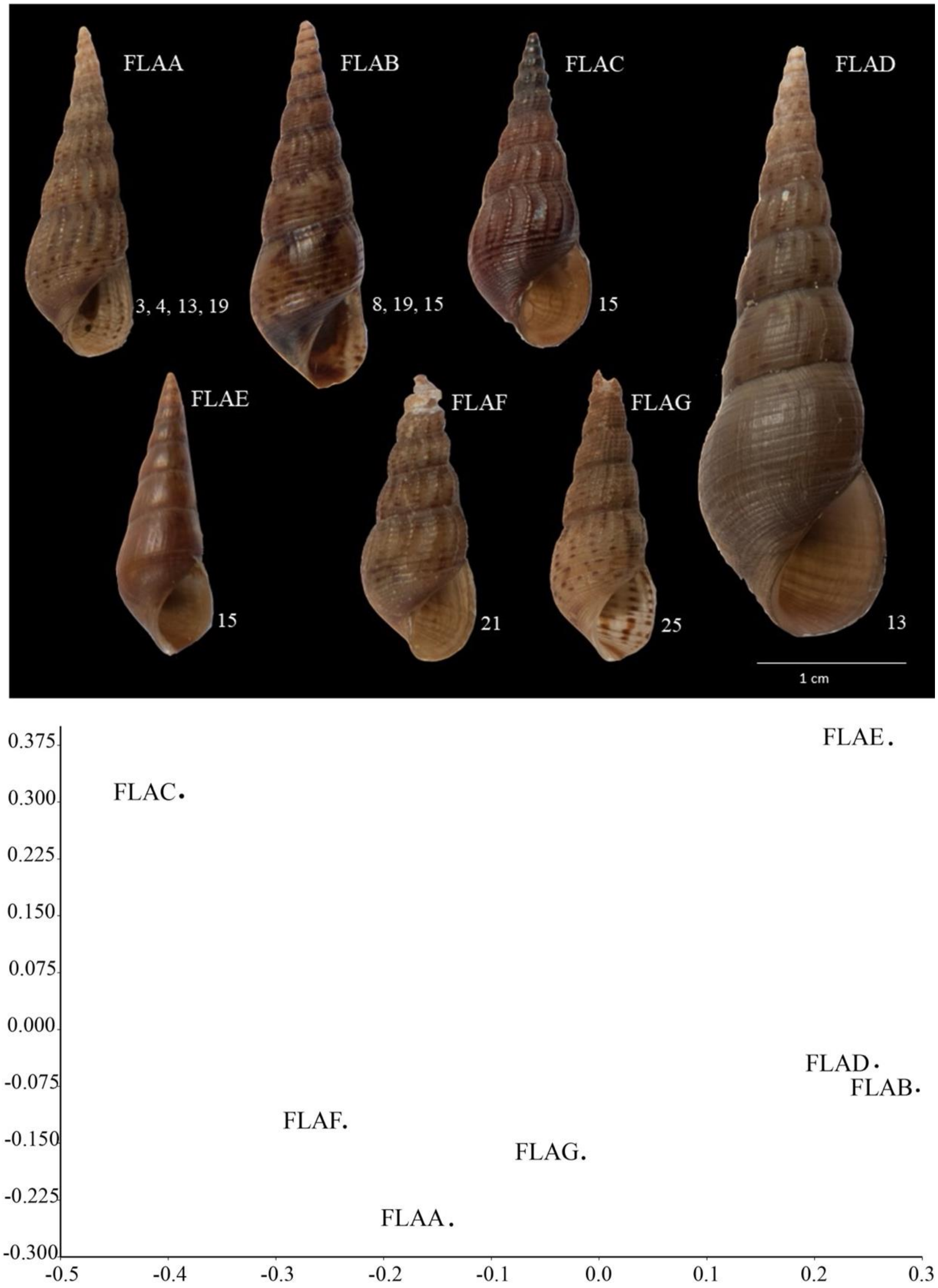

Figure 2. (Upper) Images of Florida shell morphs (FLAA – FLAG). Site locations of each morph are included. Scale bar = 1 cm. (Lower). Non-dimensional scaling analyses (NDS) of Florida morphs (FLAA – FLAG). Stress = 0.0602

Table 4. Summary of Florida shell morph characteristics. Shell colors, patterns, columella patterns, shape, and sculpture characters used to generate a data matrix to determine shell morphs. Characters are described in Facon et al. (2003).

Morphs	background color			color patterns				columellar band		general shape		sculpture		
	IN	TI	HE	DO	SP	SO	HO	SH	SC	CO	RO	GR	RI	RD
**FLAA**	2	2	0	2	3	1	2	3	2	2	2	2	3	3
**FLAB**	3	2	0	2	2	3	1	3	3	1	2	1	0	1
**FLAC**	4	3	0	0	0	0	0	0	0	2	3	3	3	3
**FLAD**	2	1	0	2	2	2	3	1	0	1	1	1	0	0
**FLAE**	3	1	0	0	0	0	0	2	2	1	1	0	0	0
**FLAF**	2	2	1	2	2	3	1	1	0	2	3	2	3	2
**FLAG**	2	2	1	2	2	2	2	2	2	2	2	2	2	2

Character Explanation

Background color:

IN-intensity of the shell background color: (1) very pale (2) pale (3) medium (4) dark

TI-background tint of the shell: (1) yellow to brown (2) greenish (3) orange to reddish (4) white (5) black

HE-heterogeneity of the background color among different parts of a shell whorl: (0) homogenous (1) distinctly darker band below the suture

Color pattern:

DO-overall density of reddish-brown color patterning on the whole shell, except one just below sutures: (0) no patterning (1) medium (2) dense

SP-type of patterning, expressed as the proportion of spots vs. flames (0) only flames (1) more flames than spots (2) more spots than flames (3) only spots

SO-size of individual spots/flames: (1) small spots/marrow flames (2) medium (3) large spots/wide flames

HO – heterogeneity of color patterning among different parts of the whorl: (1) homogenous (2) slightly different patterns just below sutures (3) strongly different patterns in the subsutural zone compared to those on the rest of the shell

Columellar band:

SH-size of the columellar band, when present: (1) narrow, (2) medium, (3) wide.

SC-size of the dark band when present: (1) narrow (2) medium (3) wide

General shape:

CO-conicity of the shell: (1) acute (2) medium (3) blunted cone

RO-roundness of the body whorl: (1) flat (2) slightly rounded (3) well-rounded

Sculpture:

GR-spiral cords/grooves: (0) absent (1) shallow grooves/weakly pronounced cords (2) intermediate (3) very deep grooves/strongly pronounced cords

Ri-density and width of axial ribs: (0) none (1)a few marrow ribs (2) a few large ribs (3) many narrow ribs

RD-depth of axial ribs when present (1) shallow (2) medium (3) deep

DISCUSSION

In our study, 49 sequenced individuals along with the 36 Genbank sequences resulted in a robust genetic dataset for us to evaluate invasion patterns of *M. tuberculata* ~~(Fig.1)~~. Similar to other studies, we found molecular evidence to support a range of putative source populations ~~(Fig. 1)~~ [9,10,17,18,34]. However, shell morphology as a tool to evaluate invasion dynamics was equivocal for identifying source populations ~~(Fig. 2)~~. Our results suggest that independent introductions are likely the primary mechanism of dispersal of *M. tuberculata* in Florida.

Genetic Diversity and Invasion Patterns

We identified nine haplotypes with a high degree of genetic structuring based on the numbers of polymorphic sites, haplotype diversity, and nucleotide diversity ~~(Table 3)~~. The spatial distribution of these identified haplotypes was random (see Mantel results above) which is like findings from Samadi et al. [20] and Facon et al. [17]. There are several possible mechanisms to explain these observed patterns but centrally we suggest that multiple invasions rather than local-scale range expansion structure snail demographic in Florida.

Our findings of seven haplotypes that were identical to sequences for the 'World' dataset are similar to findings by Facon et al. [17] that showed multiple lineages in invaded systems in South and Central America. Likewise, Harding et al. [18] suggested multiple invasions in central Texas based on the occurrence of 3 distinct haplotypes. The invasion patterns of the parthenogenetic New Zealand mud snail (*Potamopyrgus antipodarum*) also demonstrate this general pattern where only two of a possible 16 haplotypes occurred in an invaded region [36]. These studies plus our results provide evidence for the importance of multiple invasions in structuring phylogeographic patterns in invaded regions.

In addition to the haplotype evidence, historical records of *M. tuberculata* distributions in Florida are useful for understanding invasion dynamics (Table 5). Over a 5-year collection period (1966-1971), snails were found in multiple regions suggesting that these snail populations were established years prior to collection dates. Collection records for our Sites 13 and 15 date back to the early 1970s [23] while the first collections from our Site 4 were first made in 2003 by Chadwick et al. [22]. Although populations may have been established earlier than the late 1960s, it is difficult to determine if snails collected in this study are legacies of original invaders (e.g. parthenogenetic daughters) or from subsequent invasions. We know from our study that snails can be eliminated as seen from two sites where records of invasion exist but only shells were recovered ~~(Fig. 2, Table 5)~~. *Melanoides tuberculata* is known to be susceptible to mortality when water temperatures are sustained for several days at below 15°C [5] and this has been observed in southern Florida by Wingard et al. [21]. Therefore, the findings of multiple haplotypes in some locations and observations of snail extirpations from other localities further point to invasion processes rather than local-scale range expansions.

Table 5. Earliest locality records from 1966 to 1971 of *M. tuberculata* from waterbodies throughout Florida. Locations where snails were collected during this study show the site number for live individuals collected, ND for no snails recovered, and dead shells for sites where shells were collected. Records were queried from databases held by: FLMNH is Florida Museum of Natural History. ANS is the U.S. Geological Survey Nonindigenous Aquatic Species Database (https://nas.er.usgs.gov/).

Locality	Latitude	Longitude	Year	Collector	Database	This study
Lake Osceola, Miami	25.7183	-80.2793	1966	Clench, W.	FLMNH	ND
pond, Miami, FL	25.9420	-80.1553	1968	Clench, W.	FLMNH	Dead shells
Lithia Springs, Tampa	27.8663	-82.2308	1969	FLMNH	FLMNH	
Hillsborough River, Tampa	27.9668	-82.4747	1969	FLMNH	FLMNH	
Hillsborough River, Tampa	28.1039	-82.2781	1969	Clench, W.	FLMNH	
Purity Springs, near Tampa	28.0219	-82.4620	1969	Thompson, F.	ANS	
Retention Ponds, Melbourne	28.0836	-80.6081	1970	Thompson, F.	FLMNH	Site 19
Rainbow Springs	29.0697	-82.4272	1970	Athearn, H.	NCSM	
Middle River Canal,	26.1727	-80.2289	1971	Russo, T.	FLMNH	Site 15
Pompano Canal, near Ft. Lauderdale	26.2315	-80.1492	1971	Russo, T.	FLMNH	Site 13
Gulf Stream, near Ft. Lauderdale	26.3155	-80.0825	1971	Reinert, J.	ANS	
Miami R., Miami	25.7793	-80.2087	1971	Russo, T.	ANS	
Fish Eating Creek, near Lakeport	26.9309	-81.2881	1971	Athearn, H.	NCSM	Dead shells

Phylogenetic reconstruction allowed us to identify lineage origins of the 'Florida' haplotypes ~~(Fig. 1)~~. Both clades 1 and 2 had sequences from Southeast Asia which shared most Florida haplotypes. Specifically, haplotypes *a* through *d* and *g* shared haplotypes of individuals from Malaysia [16]. Chiu et al. [16] also included a phylogenetic reconstruction using 16s rDNA which suggests that haplotypes from other studies of invaded regions (Caribbean, Central America, South America, Africa, and Miami, Florida) originated from Malaysia [10,17,34]. Our results further corroborate this phylogeographic pattern except for haplotype *e* which matched individuals collected in Iran (Nasibi, unpublished data). Although these sequences are in clade 1, they separate into their own sub-clade that does not include sequences from Southeast Asia (~~Fig~~. ~~1~~ - It is likely that haplotype *e* is from the Middle East or other regions of Asia.).

Mechanisms of invasion of *M. tuberculata* are suggested as links to the aquarium trade [6,7,9,37]. The fact that these seven haplotypes originated primarily from Southeast Asia suggests that the invasion route is primarily from Malaysia, as has been reported in other studies [10,17,18] again points to invasions being facilitated by the aquarium trade [8,9,38,39]. In Florida, the release of invasive species is a pervasive and a well-documented environmental problem [40].

The establishment of 9 distinct haplotypes demonstrates that gene flow is limited. The presence of multiple haplotypes at 6 of the 11 sites, even when only three or four individuals were sequenced suggests that these populations are female clonal. In fact, we collected only one male during this study. Parthenogenetic reproduction is the primary reproductive trait that has allowed *M. tuberculata* to succeed in its invasive ranges [41]. Interestingly, we did see some differences in genetic variation between 'Florida' and 'World' haplotypes which may suggest that invasive haplotypes in Florida have diverged from their lineage origins. This was seen for Florida haplotypes *h* (site 4) and *i* (site 13) where these haplotypes were not identified in the 'World" dataset. Both sites 4 and 13 included haplotype *g* which perhaps diverged into haplotype *h* (site 4) and *i* (site 13), respectively through sexual reproduction as these snails may be descendants of invasion events from ~70 years ago. The long-term colonization possibilities for these populations can lead to mutations due to rare sexual reproduction events given the long timespan. Alternatively, it is still possible that haplotypes *h* and *i* are haplotypes not currently available on GenBank. As such, additional molecular markers are needed to evaluate these alternative hypotheses.

Shell Morphology

Melanoides tuberculata is well known to be represented by a wide range of shell morphs, however, these variations have been shown to be poorly linked to genetic differentiation [15,17]. Duggan and Knox [9] point out in their study in New Zealand that shell characteristics were not reliable for identifying lineage origins despite strong genetic evidence. Similarly, undistinguishable shell morphologies between native and invasive *M. tuberculata* in African rift lakes provide for “camouflaged invasions” in the region [10]. However, previous studies of the relationship between shell form and genetic diversity have shown that shell forms can align with snail haplotype [20] in invaded regions of central and south America. Additionally, Facon et al. [17] suggested that shell morphs tended to correlate with genetic lineages.

For our study, we found mixed alignment between haplotype and shell morphs. For example, shell morphs FLAA and FLAD which were close together in morpho-space (Fig. 2) were from haplotypes from different clades (Fig.1). Conversely, haplotypes *a*, *c*, and *d* (clade 1) and haplotypes *g*, *h* (clade 2) converged on a different shell morph (FLAB and FLAA, respectfully). Additionally, we found different shell morphs for the same haplotype (i.e., haplotypes *a* and *g*) from different sampling locations. These results support ideas present in Duggan and Knox [9] and suggest that morphological shell characteristics lack the resolution to discriminate among *M. tuberculata* taxa and their likely lineage origins.

Conclusions

While we found no spatial correlations of haplotypes, region of origin, or shell morphs in our study, we were able to show that currently in Florida there are 9 discreet haplotypes. The haplotypes were shown to have likely origins from several regions globally. While we are unable to definitively identify pathways or modes of invasion it is evident that multiple invasions have

occurred in the >50 years since these snails were first identified in Florida waters. We suggest that independent introductions occur state-wide that the likely mechanism of invasion and local-scale range expansions are related to aquarium trade activities.

Acknowledgments

Jessica Wooten and Nicholas (Reed) Alexander, Piedmont College, Georgia performed the DNA extraction and DNA amplification.

Funding Declaration

Funding was provided by United States Fish and Wildlife Service, Gulf States Marine Fisheries Division (project ID: FWS-800-037-2016-JSU) and the McCluer Scholar Award given to L. Tolley-Jordan by the School of Science, Jacksonville State University.

Author Contributions

Conceptualization: LTJ and MAC; Data curation: LTJ; Method and Analysis: All authors; Writing – original draft: LTJ; Writing – review & editing: All authors

References

1. Tolley-Jordan LR, Chadwick MA. *Centrocestus formosanus* Nishigori (Asian gill-trematode). A Handbook of Global Freshwater Invasive Species Earthscan, New York NY, USA. 2012; 401–419.

2. Weir SM, Salice CJ. High tolerance to abiotic stressors and invasion success of the slow growing freshwater snail, *Melanoides tuberculatus*. Biol Invasions. 2012;14: 385–394. doi:10.1007/s10530-011-0084-x

3. Roessler MA, Beardsley GL, Tabb dc. New records of the introduced snail, *Melanoides tuberculata* (mollusca: Thiaridae) in south Florida. Florida Scientist. 1977;40: 87–94.

4. Chaniotis BN, Butler JM, Ferguson FF, Jobin WR. Bionomics of *Tarebia granifera* (Gastropoda: Thiaridae) in Puerto Rico, an Asiatic vector of *Paragonimiasis westermani*. Caribbean Journal of Science. 1980;16: 81–90.

5. Mitchell AJ, Brandt TM. Temperature Tolerance of Red-Rim Melania *Melanoides tuberculatus*, an Exotic Aquatic Snail Established in the United States. Transactions of the American Fisheries Society. 2005;134: 126–131. doi:10.1577/FT03-178.1

6. Madsen H, Frandsen F. The spread of freshwater snails including those of medical and veterinary importance. Acta Tropica. 1989;46: 139–146. doi:10.1016/0001-706X(89)90030-2

7. Pointier JP, Delay B. Spread of the introduced freshwater snail *Melanoides tuberculata* (Müller, 1774) on the island of Guadeloupe, French West Indies (Prosobranchia, Thiaridae). Haliotis (Paris). 1995;24: 109–116.

8. Ng TH, Tan SK, Wong WH, Meier R, Chan S-Y, Tan HH, et al. Molluscs for Sale: Assessment of Freshwater Gastropods and Bivalves in the Ornamental Pet Trade. PLOS ONE. 2016;11: e0161130. doi:10.1371/journal.pone.0161130

9. Duggan I, Knox M. The origins of *Melanoides tuberculata* (Müller, 1774) in New Zealand's aquarium trade and non-indigenous population. AI. 2022;17: 393–401. doi:10.3391/ai.2022.17.3.04

10. Van Bocxlaer B, Clewing C, Mongindo Etimosundja J-P, Kankonda A, Wembo Ndeo O, Albrecht C. Recurrent camouflaged invasions and dispersal of an Asian freshwater gastropod in tropical Africa. BMC Evolutionary Biology. 2015;15: 33. doi:10.1186/s12862-015-0296-2

11. POINTIER JP, INCANI RN, BALZAN C, CHROSCIECHOWSKI P, PRYPCHAN S. Invasion of the rivers of the littoral central region of Venezuela by *Tarebia granifera* and *Melanoides tuberculata* (Mollusca: Prosobranchia: Thiaridae) and the absence of *Biomphalaria glabrata*, snail host of *Schistosoma mansoni*. Nautilus (Philadelphia, PA). 1994;107: 124–128.

12. Hotchkiss ER, Hall RO. Linking calcification by exotic snails to stream inorganic carbon cycling. Oecologia. 2010;163: 235–244. doi:10.1007/s00442-009-1536-1

13. Tolley-Jordan LR, Owen JM. Habitat influences snail community structure and trematode infection levels in a spring-fed river, Texas, USA. Hydrobiologia. 2008;600: 29–40. doi:10.1007/s10750-007-9173-3

14. Karatayev AY, Burlakova LE, Karatayev VA, Padilla DK. Introduction, distribution, spread, and impacts of exotic freshwater gastropods in Texas. Hydrobiologia. 2009;619: 181–194. doi:10.1007/s10750-008-9639-y

15. Sørensen L, Jørgensen A, Kristensen TK. Molecular diversity and phylogenetic relationships of the gastropod genus *Melanoides* in Lake Malawi. African Zoology. 2005;40: 179–191. doi:10.1080/15627020.2005.11407317

16. Chiu Y-W, Gan Y-C, Kuo P-H, Hsu K-C, Tan M-S, Ju Y-M, et al. Mitochondrial Genetic Diversity of the Freshwater Snail *Melanoides tuberculata*. Biochem Genet. 2019;57: 323–337. doi:10.1007/s10528-018-9892-3

17. Facon B, Pointier J-P, Glaubrecht M, Poux C, Jarne P, David P. A molecular phylogeography approach to biological invasions of the New World by parthenogenetic Thiarid snails. Molecular Ecology. 2003;12: 3027–3039. doi:10.1046/j.1365-294X.2003.01972.x

18. Harding S, Rodriguez D, Jackson J, Huffman D. Genetic and morphological variation is associated with differences in cold-water tolerance and geographic expansion among invasive snails (*Melanoides tuberculata*) in central Texas. bioRxiv; 2019. p. 2019.12.20.884866. doi:10.1101/2019.12.20.884866

19. Livshits G, Fishelson L, Wise GS. Genetic similarity and diversity of parthenogenetic and bisexual populations of the freshwater snail *Melanoides tuberculata* (Gastropoda: Prosobranchia). Biological Journal of the Linnean Society. 1984;23: 41–54. doi:10.1111/j.1095-8312.1984.tb00805.x

20. Samadi S, Mavárez J, Pointier J-P, Delay B, Jarne P. Microsatellite and morphological analysis of population structure in the parthenogenetic freshwater snail *Melanoides tuberculata*: insights into the creation of clonal variability. Molecular Ecology. 1999;8: 1141–1153. doi:10.1046/j.1365-294x.1999.00671.x

21. Wingard GL, Murray JB, Schill WB, Phillips EC. Red-Rimmed Melania (*Melanoides tuberculatus*) - A snail in Biscayne National Park, Florida - Harmful invader or just a nuisance? Fact Sheet. U.S. Geological Survey; 2008. Report No.: 2008–3006. doi:10.3133/fs20083006

22. Chadwick MA, Thiele JE, Huryn AD, Benke AC, Dobberfuhl DR. Effects of urbanization on macroinvertebrates in tributaries of the St. Johns River, Florida, USA. Urban Ecosystems. 2012;15: 347–365.

23. Russo, T.N. Discovery of the gastropod snail *Melanoides* (Thiara) *tuberculata* (Müller, 1774) in Florida. Florida Scientist. 1974; 212–213.

24. Tolley-Jordan L, Chadwick M, Triplett J. New records of digenetic trematodes infecting *Melanoides tuberculata* (Müller, 1774) in Florida, USA. Bioinvasions Records. 2022;11: 149–164.

25. Schrader C, Schielke A, Ellerbroek L, Johne R. PCR inhibitors – occurrence, properties and removal. Journal of Applied Microbiology. 2012;113: 1014–1026. doi:10.1111/j.1365-2672.2012.05384.x

26. Folmer O, Black M, Hoeh W, Lutz R, Vrijenhoek R. DNA primers for amplification of mitochondrial cytochrome c oxidase subunit I from diverse metazoan invertebrates. Mol Mar Biol Biotechnol. 1994;3: 294–299.

27. Kumar S, Stecher G, Li M, Knyaz C, Tamura K. MEGA X: Molecular Evolutionary Genetics Analysis across Computing Platforms. Mol Biol Evol. 2018;35: 1547–1549. doi:10.1093/molbev/msy096

28. Rozas J, Ferrer-Mata A, Sánchez-DelBarrio JC, Guirao-Rico S, Librado P, Ramos-Onsins SE, et al. DnaSP 6: DNA Sequence Polymorphism Analysis of Large Data Sets. Molecular Biology and Evolution. 2017;34: 3299–3302. doi:10.1093/molbev/msx248

29. Ronquist F, Teslenko M, van der Mark P, Ayres DL, Darling A, Höhna S, et al. MrBayes 3.2: Efficient Bayesian Phylogenetic Inference and Model Choice Across a Large Model Space. Systematic Biology. 2012;61: 539–542. doi:10.1093/sysbio/sys029

30. Huelsenbeck JP, Rannala B. Frequentist Properties of Bayesian Posterior Probabilities of Phylogenetic Trees Under Simple and Complex Substitution Models. Systematic Biology. 2004;53: 904–913. doi:10.1080/10635150490522629

31. Mason-Gamer RJ, Kellogg EA. Testing for Phylogenetic Conflict Among Molecular Data Sets in the Tribe Triticeae (Gramineae). Systematic Biology. 1996;45: 524–545. doi:10.1093/sysbio/45.4.524

32. Wilcox TP, Zwickl DJ, Heath TA, Hillis DM. Phylogenetic relationships of the dwarf boas and a comparison of Bayesian and bootstrap measures of phylogenetic support. Molecular Phylogenetics and Evolution. 2002;25: 361–371. doi:10.1016/S1055-7903(02)00244-0

33. Swofford DI. PAUP : phylogenetic analysis using parsimony (and other methods), 4.0 beta. http://paup.csit.fsu.edu/. 2002 [cited 29 Sep 2022]. Available: https://cir.nii.ac.jp/crid/1573387450415079808

34. Genner MJ, Michel E, Erpenbeck D, De Voogd N, Witte F, Pointier J-P. Camouflaged invasion of Lake Malawi by an Oriental gastropod. Molecular Ecology. 2004;13: 2135–2141. doi:10.1111/j.1365-294X.2004.02222.x

35. Hammer O, Harper DAT, Ryan PD. PAST: Paleontological Statistics Software Package for Education and Data Analysis.

36. Städler T, Frye M, Neiman M, Lively CM. Mitochondrial haplotypes and the New Zealand origin of clonal European *Potamopyrgus*, an invasive aquatic snail. Molecular Ecology. 2005;14: 2465–2473. doi:10.1111/j.1365-294X.2005.02603.x

37. Scholz T, Aguirre-Macedo ML, Salgado-Maldonado G. Trematodes of the family Heterophyidae (Digenea) in Mexico: a review of species and new host and geographical records. Journal of Natural History. 2001;35: 1733–1772. doi:10.1080/00222930152667087

38. Rixon CAM, Duggan IC, Bergeron NMN, Ricciardi A, Macisaac HJ. Invasion risks posed by the aquarium trade and live fish markets on the Laurentian Great Lakes. Biodivers Conserv. 2005;14: 1365–1381. doi:10.1007/s10531-004-9663-9

39. Yanai Z, Dayan T, Mienis H, Gasith A. The pet and horticultural trades as introduction and dispersal agents of non-indigenous freshwater molluscs. MBI. 2017;8: 523–532. doi:10.3391/mbi.2017.8.4.07

40. Della Venezia L, Samson J, Leung B. The rich get richer: Invasion risk across North America from the aquarium pathway under climate change. Diversity and Distributions. 2018;24: 285–296. doi:10.1111/ddi.12681

41. Rader RB, Belk MC, Keleher MJ. The Introduction of an Invasive Snail (*Melanoides tuberculata*) to Spring Ecosystems of the Bonneville Basin, Utah. Journal of Freshwater Ecology. 2003;18: 647–657. doi:10.1080/02705060.2003.9664007

Click here to access/download
Supporting Information
Supplementary Genbank table.xlsx
