# Supplementary material for: DNA barcoding indicates multiple invasions of the freshwater snail Melanoides tuberculata sensu lato in Florida
Source: PLoS One. 2023 Dec 22;18(12):e0292164. doi: 10.1371/journal.pone.0292164 (PMC10745163; doi:10.1371/journal.pone.0292164)
Supplement: Attachment — Submitted filename: Comment-PLOS10.docx [file pone.0292164.s001.docx]

After reviewing the manuscript titled “DNA barcoding indicates multiple invasions of the freshwater snail *Melanoides tuberculata* in Florida”, the authors have investigated the patterns of dispersal of snails in Florida through phylogenetic reconstruction including sequences from globally distributed snails using mitochondrial COI markers. This report covers important new ground for resolving the introduction of historical phylogeography and population genetic structure unique to Florida freshwater snails in relation to the world. As such it warrants publication in PLOS ONE as an important regional reference for sustained management and protection of evolutionary biodiversity in an area of progressive invasive disturbance due to anthropogenic impacts. The manuscript would benefit by integrating the different categorical results across biomarkers into a summary Figure 3 that illustrates the main introduction historical scenario described near the end of the Discussion and proposed by the authors as reflected by the title of the study. This should be mapped in historical detail onto the geographic region of Florida vs. the world and not just be summarized as an abstract conceptual diagram as in Fig. 1. Although this paper is well written, it wears some mistakes in data analysis and interpretation. The abstract is dreadful and looks as though it has been written by an inexperienced team member, without scrutiny by the rest of the team. The abstract will need to be rewritten to achieve clarity and give the reader the confidence that the manuscript is going to be worth reading. The described aims are not clear, and any hypotheses were proposed. In the introduction section, the author must have revised and described the geology of Florida in detail. It will help the readers understand the dispersed primarily through independent invasions, range expansion of populations through connected waterways, or a combination of both. In the Methodology section, there are some mistakes in the phylogenetic analysis. We suggested that the most appropriate model was used by SMS: Smart Model Selection in PhyML. We strongly suggested that the author should reconstruct the network topology that has generated a given invasive scenario. Their results need to be discussed in perspective. Despite the comprehensive data presented the results and discussion were not profoundly discussed. Figure 1, A map with more illustrations is needed for the paper. The current map only has the sampling locations’ initials on it. It is really difficult for someone not familiar with the river system in the region of Florida to understand this paper and get the main idea of the paper. While reading the manuscript we encountered several issues that need the most careful attention from the authors. Some issues are semantic/conceptual others are technical. We tried making my review as constructive as possible and hoped the authors find it useful. In my opinion, this manuscript does not meet the criteria for publication and must therefore be minor revised.

I have no minor comments.
