# Supplementary material for: DNA barcoding indicates multiple invasions of the freshwater snail Melanoides tuberculata sensu lato in Florida
Source: PLoS One. 2023 Dec 22;18(12):e0292164. doi: 10.1371/journal.pone.0292164 (PMC10745163; doi:10.1371/journal.pone.0292164)
Supplement: Attachment — Submitted filename: Responses to Reviewers.docx [file pone.0292164.s003.docx]

“DNA barcoding indicates multiple invasions of the freshwater snail *Melanoides tuberculata* in Florida”.

Response to Journal Requirements

1. We have prepared all documents to match journal style requirements.

2. Permits. Oddly enough, no permits are required to sample invasive snails in Florida. Collection and transport of this species is not federally regulated, or state regulated in Florida.

3. All genetic sequences are accessioned on GenBank and obtaining a DOI.

4. The update map submitted in this revision was generated in R and does not violate any copyright laws.

**Responses to reviewers.**

First off, we very much appreciate the extensive time and effort of the reviewers in providing meaningful suggestions needed to publish these findings. The reviewers will find that their comments are incorporated to provide clear study aims, appropriate methodologies, and meaningful results. As such, this manuscript is re-written and now provides strong evidence of multiple invasions of *M. tuberculata* in Florida.

*Author Response to Reviewer 1*:

The introduction has been significantly revised to clarify the context of historical data and hydrology. We added extensive information in the introduction to provide a more detailed context of relevant hydrologic features that may be important in the dispersal of these snail in Florida. The locality data from historic collections is now found in table 1 and cities listed in the table are found on an updated map (Figure 1) that correspond to the sites sampled in this study. In addition, the aims of the study are explicitly stated as alternative hypotheses tested based on a phylogeographic framework using the barcoding gene COI. The methods were updated to provide robust methodologies for testing hypotheses stated in the introduction. For instance, the updated methodology on analyses of genetic diversity addresses issues with network topology and other inconsistencies. Also, the methodologies used in phylogenetic reconstruction were extensively updated which included more taxa putatively listed as M. tuberculata and other Thiarids to reflect taxonomic issues with this species and more genetic structure to test invasion hypotheses. These new approaches are reflected in the organization of both the methods and results. These changes are reflected in the results which includes more tables and statistical tests on haplotype diversity that provide evidence to support the hypothesis of multiple invasions. The inclusion of additional sequences of M. tuberculata and other Thiarids made a much more informative tree to explain relationships between haplotypes recovered in Florida within a global framework. The discussion is significantly strengthened as the significance of findings are explicitly stated and corroborated by other studies that reported similar findings based on using barcoding genes to understand invasion dynamics. Given this significant overhaul of the manuscript, the abstract now reflects updated aims, methods, results, and interpretation in a clear and concise manner.

*Author Response to Reviewer 2*:

All comments left in the text are addressed due to the complete rewrite of the manuscript in all sections. We agree that the previous version did not state hypotheses, provide methodologies, or results to test hypotheses which ultimately led to a discussion that did not provide new insight into the dispersal of this species. Further, statements about aquarium trade as the most likely vector of invasion had little support. In this revision, we took great care in strengthening the introduction to show the significance of aquarium trade in movement of taxa into and out of Florida. We also provided evidence to show that *M. tuberculata* is one of the most sold freshwater snail species in the aquarium trade both with online hobbyist and pet stores. We hope that these details strengthen our implications of the pet trade in the dispersal of these snails in Florida. Given the extensive updates to this revision we feel that the significance of the work is clearly shown in the discussion and abstract.

*Author Response to Reviewer 3*:

We very much appreciate that Dr. Hayes noticed the unusual shell forms found in Florida as possible species of *Stenomelania*. Prior to this review, we did not consider this possibility as no published reports of established populations of *Stenomelania* in the US were recovered. There is a very strong likelihood that *Stenomelania* *spp*. are in Florida, and elsewhere, given the trade of Black Mudalia (*Stenomelania spp.* most likely) sold by aquarists on Amazon and other online markets. As photo vouchers are not always supplied with locality records, it is difficult to know the extent, if any, of the introductions of other Thiaridae spp. Further, we also were unaware of the pervasive issues in taxonomy of many species of Thiarids and that the separate clades of *M. tuberculata* represented polyphyly. Inclusion of *Stenomelania spp*., *Tarebia granifera*, and *Melanoides polymorpha* in this data set yielded a far better perspective on reasons for such strong genetic divergence between the clades of putative *M. tuberculata*. Our findings showed that all individuals sequenced from Florida in this study are within the two putative clades of *M. tuberculata*. As such, we did not make any claims that these individuals are in another genus. However, we recognize the constraints of our findings due to limited numbers of individuals sequenced per site and limited numbers of sampling sites to give a full picture of the invasive lineages in Florida. At this time, we did not have the funding to include additional molecular markers to add to the results of this study and none of the authors are in Florida to collect additional samples. Regardless the additional GenBank sequences provided a more robust dataset for both population genetics and phylogenetic analyses. These updates added the necessary context to evaluate the invasion patterns in based on the samples collected in this study.
