# Supplementary material for: DNA barcoding indicates multiple invasions of the freshwater snail Melanoides tuberculata sensu lato in Florida
Source: PLoS One. 2023 Dec 22;18(12):e0292164. doi: 10.1371/journal.pone.0292164 (PMC10745163; doi:10.1371/journal.pone.0292164)
Supplement: Attachment — Submitted filename: comments-PLOSONE06.docx [file pone.0292164.s004.docx]

After reviewing the manuscript titled " DNA barcoding indicates multiple invasions of the freshwater snail *Melanoides tuberculata* in Florida", the authors have investigated the patterns of dispersal of snails in Florida through phylogenetic reconstruction including sequences from globally distributed snails using mitochondrial COI markers. I feel that the manuscript has been significantly improved and satisfied with previous revisions. The whole manuscript and abstract are well-written and organized; hypothesis-test is obviously well defined. The data and analysis generally appear to be sound, the results are clear and interesting. After careful consideration, I feel that this manuscript will likely be suitable for publication.
