# Supplementary material for: DNA barcoding indicates multiple invasions of the freshwater snail Melanoides tuberculata sensu lato in Florida
Source: PLoS One. 2023 Dec 22;18(12):e0292164. doi: 10.1371/journal.pone.0292164 (PMC10745163; doi:10.1371/journal.pone.0292164)
Supplement: Attachment — Submitted filename: PONE_D_22-27-150_Responses.docx [file pone.0292164.s005.docx]

4 September 2023

**PLOS ONE Editorial Board**
**Handling Editor:** Dr Hudson Alves Pinto

On behalf of my co-authors, pleased consider the attached revised manuscript now titled: “**DNA barcoding indicates multiple invasions of the freshwater snail *Melanoides tuberculata sensu lato* in Florida**” (PONE-D-22-27150) for publication by the Editorial Board at PLOS ONE.

This original research article detailed invasion dynamics of *Melanoides tuberculata*, a freshwater snail that has successfully invaded freshwaters worldwide, including Florida, USA. The critiques of the revision have improved the paper, especially related to issues about the unresolved taxonomy of our study animal. Updates to the manuscript are detailed below and in the tracked-changed version of the submission.

We appreciate your consideration of this revised manuscript, and we look forward to receiving insights from the reviewers and future publication of this important contribution.

Sincerely,

Michael A Chadwick

Corresponding author

[michael.chadwick@kcl.ac.uk](mailto:g.lagod@pollub.pl)

**Response to the editor’s general comments:**

**New Title:** “DNA barcoding indicates multiple invasions of the freshwater snail *Melanoides tuberculata sensu lato* in Florida”

We agree that the previous draft of our manuscript did not emphasize the likelihood that multiple species of Thiarids currently identified as *Melanoides* in GenBank have invaded Florida. As such, we addressed this topic in two ways. First, we changed *Melanoides tuberculata* to *M. tuberculata sensu lato* in the revised title and throughout the manuscript. The use of *sensu lato* is the correct epithet to show that these individuals are potentially phylogenetically polyphyletic and morphologically cryptic (*despite the fact that this paper is not intended to focus on this aspect of these invasive snails*). Regardless of this tricky taxonomy, our results showed multiple lineages of *M. tuberculata s.l.* have invaded Florida, strongly supporting our contention of multiple invasions rather than a single invasion with subsequent dispersal.

With respect to lack of detection of other species of invasive thiarids, we agree that this is problematic. However, our genetic data confirmed only the invasion of *M. tuberculata s.l.* and *Tarebia granifera* based on available GenBank information. We emphasize in the text the need for voucher specimens to be reported with locality records so that specimen identifications can be confirmed by taxonomic experts. Also, the prolific nature of the aquarium trade and online availability of additional Thiarids suggests that other genera are in the state and granular surveys are needed to detect new species.

**Comments below provide detailed responses to reviewer 3.**

**Responses to reviewer 3 general comments:**
First off, we very much appreciate the extensive time and effort of the reviewer in ensuring that clear, pointed comments on the extensive taxonomic issues of Thiarids. We have carefully addressed these comments in this revision (see below and in the track-changes version of the paper. Our previous version did not focus on the significance of taxonomic issues and invasion pathways of *Melanoides tuberculata*. As such, the previous revision did not highlight important components of documenting invasions such as voucher specimens, understanding life history characteristics, in addition to informative molecular markers. Our work clearly needed to be improved to emphasize that detecting many invasion events are difficult due to the cryptic nature of these snails and the need for taxonomic revision of this group. In addition, the likelihood that other species of Thiarids are in Florida but have been incorrectly identified (due to problematic taxonomy and shell phenotypic plasticity) are highlighted as confounding issues in understanding invasion dynamics. We stress that the inclusion of voucher specimens is critical for individuals that appear to be “*M. tuberculata*” given the high degree of phenotypic plasticity that masks invasions of other Thiarids, such as *Stenomelania s.l*. These changes reflect the complexity of investigating the invasion dynamics of cryptic snail species and we feel satisfactorily address concerns of Reviewer 3.

This new version highlights these issues with respect to invasion dynamics throughout the text as extensive changes to the abstract, introduction, and discussion were made. In this updated version, the epithet *Melanoides tuberculata* was changed to *Melanoides tuberculata sensu lato* to reflect the taxonomic issues of this species complex. Further, we included additional citations by Strong et al. 2011 that showed Paludomidae is a sister clade to Thiaridae to justify our choice of outgroup.

**Specific answers to the comments and concerns of reviewer 3:**

**Reviewer comments:** *I applaud the authors for making substantial revisions.*

**Response**: Thank you for the kind response of the considerable efforts we made in the last revision, and we feel that the paper is much improved. We appreciate the attention to detail of all reviewers and editors to improve this manuscript.

**Reviewer comment:** *Narrative is still focused on the premise that this is a single taxon introduced multiple times instead of the pattern shown of multiple species introduced*

**Response:** We agree that we missed the mark on clearly stating that this species is polyphyletic. As such, *sensu lato* appears in the title, the first sentence of the abstract, the beginning of the paragraph in the introduction. In the abstract and discussion, we refer to *M. tuberculata* as a species complex. We highlight the placement of Florida haplotypes into two clades that resulted in the invasion of a species complex into Florida- not a single species.

**Reviewer comment:** *It is clear that both Stenomelania, Melanoides, several other genera in the Thiaridae are in critical need of revision. And while it is not the place for these authors to undertake those revisions in this study, it is their obligation to place their work and interpret in the objective and accurate context based on this fundamental understanding*

**Response:** We recognize that the significance of the unresolved Thiaridae taxonomy was not emphasized in the previous drafts and took great care to highlight these issues throughout the text in this revision.

**Reviewer comment:** *The title still reflects this focus, and the discussion of the analyses are still centered around this false premise.*

**Response:** We have updated the title to include the term *sensu lato* to show that M. tuberculata is a species complex that in need of taxonomic revision.

**Reviewer comment:** *It would be much more informative if the authors more clearly revised the introduction, analyses, and interpretation to reflect the reality of confused taxonomy, multiple species introductions, and emphasized the work needed to resolve this.*

**Response:** We agree and included much more detail on the significance of taxonomic uncertainty in describing invasion patterns.

**Reviewer comment:** *The discussion could be improved by reflecting on the implications of the confused taxonomy*

**Response:** Please see updated information in the title, abstract, introduction, methods, and discussion.

**Reviewer comment:** *The discussion could be improved by reflecting on the implications of the confused taxonomy*

**Response**: We appreciate that the reviewer has read previous works about the importance of *Melanoides tuberculata s.l.* as a host for many trematode parasites, we have not covered this issue in this manuscript. We are unclear as to how to address this comment.

**Reviewer comment:** A*dditionally, they still implicate the pet-trade as the culprit, but provide no data from surveys of aquarium shops or any other data that would support this.*

**Response**: We refer to other papers to support this assertion, but we suggest, multiple times, that further researcher on introduction mechanisms are needed. Our work highlights the need for more research on the significance of the aquarium trade.

**Reviewer Comment:** *Justification for an outgroup is founded on a misunderstanding of what should be represented by an outgroup. In one section the authors state the used Tarebia granifera as the outgroup (pg 6 line 109), then later claim they selected Paludomus siamensis based on Wiggering et al. (2019), but they misspell the species on line 153 of page 8. More problematically, Wiggering et al. (2019) was addressing a fundamentally different question, about taxa in Thailand, and this outgroup is inappropriate for this analysis. Neither Tarebia or Paludomus are appropriate for outgroups in this situation, and the authors, if they examine Wiggering 2020 closely, they will see this. Thiara and additional Stenomelania species, and additional representatives are clearly needed to more fully understand the placement of taxa in Florida, and their affinities with other species across the globe.*
**Response:** We corrected the error about Tarebia as an outgroup which was an oversight in previous edits of the manuscript.. Outgroup selection was initially based on data from Wiggering 2020. Based on this comment, we included reference by Strong et al. 2011 that showed Paludomidae is sister to Thiaridae and The sequence from Wiggering 2020 was used due to the presence of a voucher specimen. We are unclear as why another outgroup(s) other than *Paludomus siamensis* is required given the fact that our phylogeny mirrors Wiggering 2020 and phylogenies of *M. tuberculata* generated in other studies.

**Reviewer Comment:** *Wiggering 2020 clearly shows that Melanoides is polyphyletic, but so is Thiara, Stenomelania, and others, but the authors have not discussed the implications of this on their interpretation of multiple introductions, or on their shell analysis.*

**Response:** In the discussion, we updated the section on Shell Morphology to reflect previous findings that phenotypic plasticity of shells within and among genera of Thiaridae is so pervasive that evidence of life histories and reproductive strategies are needed to parse taxa into different lineages. We included text highlighting the importance of voucher specimens, including soft tissues when available, are needed to identify specimens of Thiarids.

**Reviewer Comment:** *This really needs a major revision, not just adding comments reflecting this issues, but an actual interpretation and discussion that features the implications of the taxonomic problems and what it means for understanding introduction pathways.*

**Response:** We have markedly altered the text in this submission to reflect the concerns of the importance of taxonomy on documenting invasion patterns. We feel that this new version highlights the interactions of polyphyly, phenotypic plasticity, and overall taxonomic problems within and among lineages of Thiaridae complicate our understanding of invasion dynamics in this study. Further, without including references to taxonomic concerns an accurate reflection of the extent and severity of an invasion cannot be determined.
